# Microencapsulated polyphenol extracts from Georgia‐grown pomegranate peels delay lipid oxidation in salad dressing during accelerated and ambient storage conditions

**DOI:** 10.1002/fsn3.3776

**Published:** 2023-10-18

**Authors:** Boran Yang, Jinru Chen, Kevin Mis Solval

**Affiliations:** ^1^ Department of Food Science and Technology University of Georgia Griffin Georgia USA

**Keywords:** accelerated storage, ambient shelf life test, antioxidant, lipid oxidation, salad dressing

## Abstract

Lipid oxidation is a major cause of quality deterioration in salad dressings. This study evaluated the effect of incorporating microencapsulated polyphenol extracts via spray drying from pomegranate peels (MPP) to delay lipid oxidation in Italian‐style salad dressings (ISD) during accelerated (55°C) and ambient (25°C) storage conditions. ISDs, prepared at high (5000 rpm) and low (250 rpm) shear rates conditions, were formulated with unencapsulated polyphenol extracts from pomegranate peels (PPP), MPP, and/or grape seed extract (GSE). Lipid oxidation in ISDs was evaluated by measuring peroxide value (PV), iodine value (IV), and TBARS, stored in accelerated and ambient conditions for 21 days and 8 weeks, respectively. Tannis in extracts were measured via HPLC‐DAD and the total hydrolyzable tannin content of PPP and MPP was 283.09 and 427.74 (mg/g extract), respectively. Condensed tannins were not detected in PPP and MPP but were found in GSE (348.53 mg/g extract). Salad dressings prepared at high shear rates had significantly (*p* < .05) higher emulsion stability than those homogenized at low shear rates. Mixing conditions did not affect the lipid oxidative stability of IDSs. Salad dressing stored under accelerated storage had higher lipid oxidation (higher PV, lower IV, and higher TBARS) after 21 days than IDSs stored under ambient conditions for 8 weeks. ISDs prepared with MPPP showed significantly (*p* < .05) lower lipid oxidation than the other ISDs at the end of the shelf life studies. Results from the accelerated storage suggested that incorporating MPP could have extended the shelf life of IDSs by 20% compared to using unencapsulated polyphenol extracts. The study demonstrated that MPP delays lipid oxidation in ISDs during storage more effectively than unencapsulated extracts. MPP may serve as a natural and effective functional food ingredient for controlling lipid oxidation in high‐lipid and acidified foods.

## Graphical abstract

Natural phytochemicals such as polyphenols are effective antioxidants. Microencapsulated pomegranate peel polyphenol can be used as a novel food ingredient to delay lipid oxidation in salad dressings.

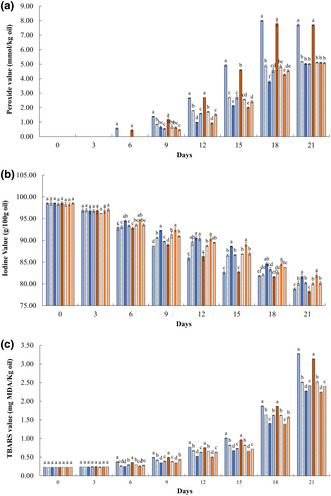

## 1 INTRODUCTION

Salad dressings are popular and versatile condiments enjoyed by many people worldwide. They are often prepared by emulsifying vegetable oils, acidifying ingredients, spices, and other additives, resulting in a variety of flavors and textures and unique styles such as Italian, Thousand Island, and French dressings (Mizani et al., 2015). Some salad dressings adopt an oil‐in‐water (o/w) or water‐in‐oil (w/o) emulsion structure, where tiny oil or water droplets are dispersed in an aqueous phase. Meanwhile, others take the form of suspensions (Arancibia et al., 2013). The physical stability of salad dressings, crucial for maintaining their structure over time, relies on several factors, including interfacial composition, emulsion droplet size, flocculation, and final phase separation (Kiokias et al., 2016; Zhang et al., 2008). Commercially available salad dressings typically come in two types: one phase and two phase. One‐phase dressings often include emulsifiers and undergo fine homogenization with high shear rates, resulting in a smooth and creamy consistency that prevents phase separation. On the other hand, two‐phase salad dressings form distinct layers of oil atop the water phase (Perrechil et al., 2010). Preparing stable and appealing salad dressings involves careful attention to processing conditions such as shear rate, temperature, and mixing time during the homogenization/mixing step. These factors are crucial to stabilizing stable salad dressings with desirable organoleptic properties (Bengoechea et al., 2009; Kim et al., 2020).

According to the U.S. Food and Drug Administration (FDA), salad dressings must contain at least 30% vegetable oil (by weight) (Ma & Boye, 2013). The most popular vegetable oils used in salad dressings are olive oil, peanut oil, and sunflower due to their great flavor, unsaturated fatty acid profile, and potential health benefits (Kaltsa et al., 2018). However, using vegetable oils with a high content of unsaturated fatty acids can challenge salad dressings' shelf life stability. This is because these oils are highly prone to lipid oxidation, which can form undesirable compounds like lipid hydroperoxides, aldehydes, ketones, and lactones (Sainsbury et al., 2016; Tseng & Zhao, 2013). According to Kiokias et al. (2016), physicochemical properties (pH and size and electrical charges of micelles), as well as processing parameters (storage temperature, homogenization conditions, oxygen, and light levels), may play roles in determining how well a salad dressing withstands lipid oxidation. To address this issue, synthetic and natural antioxidants are widely used in salad dressings to minimize or delay lipid oxidation and the formation of oxidation products that may negatively affect the taste and nutritional value of the dressings. Natural antioxidants such as fruit polyphenols and tocopherols are considered safe and effective alternatives to synthetic antioxidants such as butylated hydroxyanisole (BHA) and *tert*‐butyl hydroquinone (TBHQ) (Phisut et al., 2018). Recent studies have reported using fruit processing by‐products (peels, seeds, etc.) to develop functional foods with antioxidant and antimicrobial properties (Rosales Soto et al., 2012). This approach aligns with the current food waste reduction and sustainability initiatives, making it a promising direction for developing novel food ingredients (Pande & Akoh, 2009; Tseng & Zhao, 2013). Exciting research has shown that polyphenols obtained from pomegranate peels offer strong antioxidant benefits (Hooks et al., 2021; Pateiro et al., 2021; Shahkoomahally et al., 2021). However, directly adding polyphenol‐rich extracts to foods is technologically challenging due to their low stability during processing and storage (Santos & Meireles, 2011). When polyphenol‐rich extracts are directly added to foods may result in bitterness, astringency, and unpleasant flavors. To address this, researchers have explored novel strategies to enhance the stability and compatibility of polyphenol extracts in foods. Microencapsulation has emerged as a highly effective strategy to stabilize plant‐based bioactives with antioxidant properties (Jolayemi et al., 2021).

According to Corrigan et al. (2012), accelerated shelf‐life tests (ACSL) are cost‐effective alternatives to determine the shelf life of foods. These tests subject foods to higher storage temperatures, stronger ultraviolet light intensities, and/or prooxidants that accelerate deterioration. In the case of salad dressings, ACSL evaluations often involve exposure to temperatures between 50 and 60°C (Berton et al., 2014). Recent studies have explored incorporating phytochemicals with antioxidant properties extracted from plant by‐products to improve the oxidative stability of salad dressings during storage (Jolayemi et al., 2021; Tseng & Zhao, 2013). Nonetheless, no studies have reported the effect of shear rates and the addition of microencapsulated pomegranate peel extracts (MPP) on the oxidative stability of salad dressings. Hence, this study aimed to evaluate the influence of MPP on the physicochemical and oxidative stability of Italian‐style salad dressings homogenized at different shear rates during ACSL and ambient shelf life evaluations (AMSL).

## 2 MATERIALS AND METHODS

### 2.1 Materials

Polyphenol‐containing extract (PPP) isolated from Georgia‐grown pomegranate peels were microencapsulated by following the method previously reported by our group (Yang et al., 2022). In short, PPP was homogenized with a mixture of maltodextrin: pomegranate peel pectin (ratio 3:1, w/w) to create PPP suspensions (core: wall materials = 1:5, w/w). Then, the PPP suspensions were spray‐dried to produce microencapsulated MPP powders. A commercial grape seed extract (GSE) (Grape seed extract, Zazzee, Montebello, NY, USA) was purchased from a local store in Griffin, GA. Peanut oil, white wine vinegar, salt, red pepper flakes, garlic powder, basil leaves, and oregano were obtained from a local supermarket in Griffin, GA, USA. Iodine monochloride Wijs solution, chloroform, potassium iodide, sodium thiosulfate, starch indicator, glacial acetic acid, iso‐octane, 1, 1, 3, 3‐tetraethoxtpropane, trichloroacetic acid, and 2‐thiobarbituric acid were obtained from Fisher Scientific (Fair Lawn, NJ, USA).

### 2.2 Characterization of phenolic compounds in PPP, MPP, and GSE

Tannins and phenolic compounds in PPP, MPP, and GSE were quantified at the Watrelot Lab (Department of Food Science and Human Nutrition, Iowa State University, Ames, IA, USA) as described below.

#### 2.2.1 Tannin content

Tannins (hydrolyzable and condensed) were determined by high‐performance liquid chromatography coupled with a diode array detector (HPLC‐DAD) after directly injecting samples prepared in methanol. Punicalagin (α and β) and ellagic acid content were calculated using a commercial standard of punicalagin and ellagic acid, respectively (Mathon et al., 2019).

#### 2.2.2 Monomeric phenolic compounds

Monomeric phenolic compounds found in PPP, MPP, and GSE were characterized by direct injection of the samples prepared at 5 g/L in 13% ethanol, 5 g/L tartaric acid, pH 3.5 by HPLC‐DAD by following the method of Ritchey and Waterhouse (1999). The amounts of flavanols were reported as equivalent to (−)‐epicatechin in mg/g of extract. While quantities of ellagitannins were expressed as equivalent to β‐punicalagin in mg/g of extract.

### 2.3 Rheological properties of PPP suspensions

PPP suspensions were stirred overnight at 25°C before testing. Shear stress sweep tests were conducted in a modular compact rheometer (MCR92, Anton Paar, Graz, Austria) with a parallel plate measuring geometry (25‐mm diameter, part No. 79044, Anton Paar, Graz, Austria); using a gap of 500 μm. Then, the resultant steady shear flow curves were analyzed at 25°C, using shear rates from 1 to 100 s^−1^. Shear stress (*σ*) and apparent viscosity (*η*) were measured as a function of shear rate. Data from the flow curves were fitted to the Power law model (Equation 1) to define shear‐effected characterizations of the PPP suspensions.

(1) $$\sigma =K\dot{\gamma }n$$

where *σ* represents the shear stress (Pa), *K* is the consistency index (Pa.sn), $\dot{\gamma }$ is the shear rate (s^−1^), and *n* is the flow behavior index (dimensionless).

### 2.4 Preparation of Italian‐style salad dressing (ISD)

**FIGURE 1:**
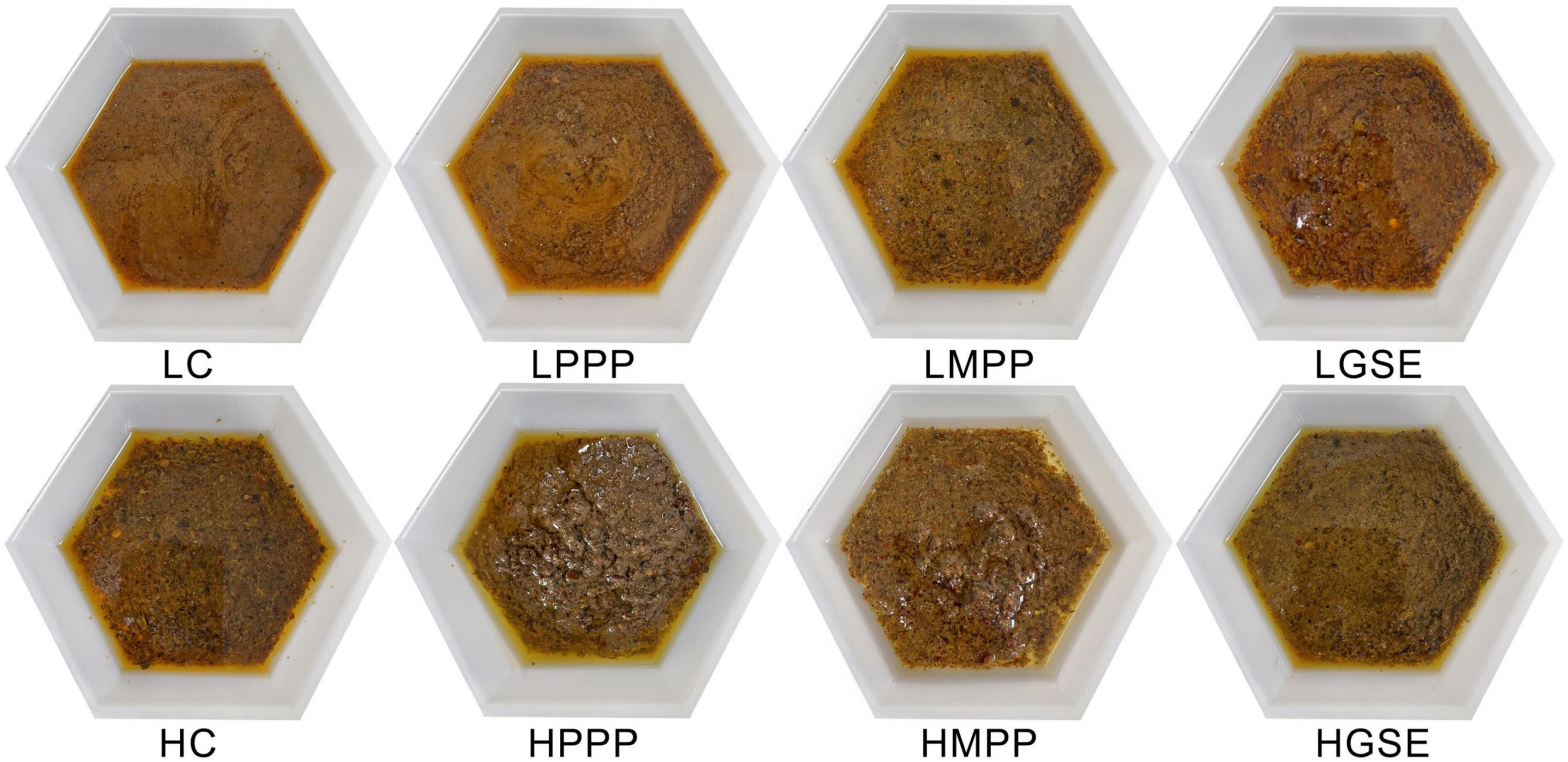
Pictures of Italian‐style homemade salad dressings (ISDs). See Table 1 for the description of LC, LPPP, LMPP, LGSE, HC, HPPP, HMPP, and HGSE.

Fresh ISDs were prepared by mixing 50 g/100 g of peanut oil, 30 g/100 g of white wine vinegar, 4 g/100 g of table salt, 2 g/100 g of garlic powder, 2 g/100 g of red pepper flakes, 1 g/100 g of basil leaves, and 1 g/100 g of oregano leaves. Afterward, either 0.5 g/100 g of PPP, 3 g/100 g of MPP powder (equivalent to 0.5 g/100 g free polyphenol‐containing extracts), or 0.5 g/100 g of GSE were added as natural antioxidants. Also, an ISD without natural antioxidants was prepared as a control. Then, the mixtures were homogenized at a shear rate of 1 *g* (LOW) or 280 *g* (HIGH) using an ultra‐high shear homogenizer (Fisherbrand 850 Homogenizer, Thermo Fisher Scientific Inc., Chicago, IL, USA) for 10 min. In total, eight different ISDs were prepared (Table 1) which were immediately characterized after production and stored under ACSL and AMSL conditions. The RGB (red, green, and blue) images of the resultant ISDS are shown in Figure 1.

**TABLE 1:** Description of Italian salad dressings (ISDs) evaluated in the study.

ISD	Mixing conditions (shear rate)	Natural antioxidant (g/100 g)		
		PPP	MPP	GSE
LC	LOW	–	–	–
LPPP	LOW	0.5	–	–
LMPP	LOW	–	3	–
LGSE	LOW	–	–	0.5
HC	HIGH	–	–	–
HPPP	HIGH	0.5	–	–
HMPP	HIGH	–	3	–
HGSE	HIGH	–	–	0.5

Abbreviations: GSE, grape seed extract; HC, ISD prepared with high shear without antioxidant; HGSE, ISD prepared with high shear with GSE; HIGH, high shear rate, 5000 rpm; HMPP, ISD prepared with high shear with MPP; HPPP, ISD prepared with high shear with PPP; LC, ISD prepared with low shear without antioxidant; LGSE, ISD prepared with low shear with GSE; LMPP, ISD prepared with low shear with MPP; LOW, low shear rate, 250 rpm; LPPP, ISD prepared with low shear with PPP; MPP, microencapsulated PPP powder; PPP, polyphenol‐containing extracts isolated from pomegranate peels.

### 2.5 Emulsifying properties of ISDs

Emulsifying capacity (EC) and emulsion stability (ES) were evaluated according to the method of Yang et al. (2018). For EC, the ISDs were centrifuged at 8000 × *g* for 12 min using a centrifuge (Model J2‐21M; Beckman Instruments Inc., Palo Alto, CA, USA). Meanwhile, for the ES, the fresh samples were held in a hot water bath (Model 2872; Thermo Fisher Scientific Inc., Marietta, OH, USA) at 80°C for at least 1 h, then the samples were centrifuged at 700 × *g* for 12 min. Afterward, the EC and ES values were calculated using Equation (2).

(2) $$\mathrm{EC}\;\text{or}\;\mathrm{ES}\;\left(\%\right)=\left(\mathrm{EL}/\mathrm{FE}\right)\times 100$$

where EL (*g*) is the mass of the resultant emulsified layer. FE is the whole mass (*g*) of the fresh samples.

### 2.6 Storage stability

Approximately 100 mL of ISDs was placed in 4 oz. regular mouth mason glass jars with metal lids (Verones Direct, Shenzhen, Guangdong, China), and stored at 55°C in an air‐forced oven (MO 1440SC, Lindberg/ Blum M, Asheville, NC, USA) for 21 days for ACSL and/or at room temperature (~25°C) in light‐proof cabinets for 8 weeks for AMSL, respectively. All ISDs were evaluated for pH, color, peroxide value (PV), iodine value (IV), and thiobarbituric acid reactive substances (TBARS) as described in Sections 2.7, 2.9. Analyses were conducted every 3 days and every 2 weeks for samples stored under ACSL and AMSL conditions, respectively.

### 2.7 pH of ISDs

Approximately, 20 mL of sample was placed in a beaker and the pH value was measured using a previously calibrated pH benchtop meter (Accumet AE150, Fisher Scientific Inc., Chicago, IL, USA).

### 2.8 Color

The color of the ISDs was measured using a Lab Scan XE Colorimeter (Hunter Associates Laboratory, Inc. Reston, VA) and the results were reported as CIE (L*, a*, and b* value). The total color difference (*ΔE*) of salad dressings was calculated from the method reported by Jiang et al. (2020) and using Equation (3):

(3) $$∆E=\sqrt{{\left(L_{0}-L_{d}\right)}^{2}+{\left(a_{0}-a_{d}\right)}^{2}+{\left(b_{0}-b_{d}\right)}^{2}}$$

where$\;L_{0}$, $\;a_{0}$, and $b_{0}$ are the values of freshly made ISDs (day 0);$\;L_{d}$,$\;a_{d}$, and $b_{d}$ are the corresponding values of the ISDs after storage for certain time intervals (days 3, 6, 9, 12, 15, 18, and 21 for ACSL; weeks 2, 4, 6, and 8 for AMSL).

### 2.9 Oxidation stability

#### 2.9.1 PV determination

The PV of ISDs was determined based on AOAC official method 965.33 (George & Latimer, 2016). Approximately 20 g of ISDs was centrifuged at 4500 × *g* for 5 min, then the top layer was collected and filtered through Whatman No. 4 filter paper (Whatman International Ltd., Maidstone, UK). Afterward, approximately 5 g of samples was dissolved in 30 mL of glacial acetic acid−isooctane (3:2, v/v). Upon addition of 0.5 mL of saturated potassium iodide solution and 30 mL of deionized water, the solutions were then titrated against 0.01 M standardized sodium thiosulfate (Na_2_S_2_O_3_) solution using 0.5 mL of 1% starch indicator until the blue color was just disappeared. The PV was calculated as shown in Equation (4).

(4) $$\mathrm{PV}=\frac{\left(S-B\right)\times C\times 1000}{2\times W}$$

where PV is reported as the millimolar peroxide per kilogram of the sample, *S* is the volume of titrant (mL) for samples, *B* is the volume of titrant (mL) for blank, *C* is the concentration of Na_2_S_2_O_3_ solution (mol/L), *W* is the mass of the samples (*g*), and 1000 is the conversion of units (g/kg).

#### 2.9.2 Quantification of IV

The IV of ISDs was calculated by following the AOAC official method 993.20 (George & Latimer, 2016). Ten grams of salad dressings was centrifuged at 4500 × *g* for 5 min, then the supernatant was collected and filtered through Whatman No. 4 filter paper. Afterward, 0.3 g of filtered samples was dissolved in 10 mL of chloroform. Next, 25 mL of Wijs solution was added and the mixture was then placed in the dark at room temperature for 1 h. Thereafter, 15 mL of 15% (w/v) potassium iodide solution and 110 mL of deionized water were added to the samples. The resultant solutions were gradually titrated against 0.1 M standardized sodium thiosulfate solution using 1 mL of 1% starch indicator until the blue color disappeared. The iodine value was calculated based on the Equation (5).

(5) $$\mathrm{IV}=\frac{\left(B-S\right)\times N\times 126.9}{W\times 1000}\times 100$$

where IV value equals to gram iodine absorbed per 100 g of sample, *B* is the volume of titrant (mL) for blank, *S* is the volume of titrant (mL) for samples, *N* is the normality of Na_2_S_2_O_3_ (mol/L), 126.9 is the molecular mass of iodine (g/mol), W is the mass of the samples (*g*), and 1000 is the conversion of units (mL/L).

#### 2.9.3 TBARS analysis

TBARS value of ISDs was determined by following the method reported by Nielsen (2017). Approximately 2 g of samples was dissolved in 10 mL of 10% trichloroacetic acid solution and centrifuged at 1200 × *g* for 5 min to collect the supernatants. Afterward, 4 mL of 0.5% 2‐thiobarbituric acid solution was added to the supernatants, and a blank (4‐mL deionized water mixed with 4 mL of 0.5% 2‐thiobarbituric acid solution) was also prepared. Then, all the samples were heated in boiling water for 40 min. After cooling to room temperature, the absorbance of samples was recorded using a Genesys 30 ultraviolet–visible spectrophotometer (Thermo Fisher Scientific Inc., Madison, WI, USA) set at *λ* = 532 nm. Quantification was based on the standard curve generated with 1, 1, 3, and 3‐tetraethoxypropane (TEP), and the result was reported as mg TEP/kg.

### 2.10 Statistical analysis

All the experiments and analyses were carried out in triplicate determinations. Means and SD of experimental results were reported, and the data were analyzed using the statistical software SAS (SAS University edition version 3.8, SAS Institute, Cary, NC, USA). The significant differences among means of experimental results were analyzed by an analysis of variance (ANOVA). A *p* value less than alpha = 0.05 was statistically significant.

## 3 RESULTS AND DISCUSSION

### 3.1 Phenolic compound analysis of PPP, MPP, and GSE

The tannin content of PPP, MPP, and GSE is shown in Table 2. The total hydrolyzable tannin content of PPP and MPP was 283.09 ± 98.81 and 427.74 ± 28.53 (mg/g extract), respectively. Condensed tannins were not detected in PPP and MPP, but were found in GSE (348.53 ± 173.09 mg/g extract), which is in line with previous work integrated by Cai et al. (2017). Canuti et al. (2020) have reported that hydrolyzable tannins, particularly ellagitannins including α‐punicalagin and β‐punicalagin, are highly effective in regulating oxidation and protecting the wine against chemical oxidation. It is important to note that the analyses were conducted in another state in the US. During storage, transportation, and preparation of the samples, the tannins in the PPP might have degraded due to exposure to environmental factors such as light, heat, and oxygen. The tannin content in MPP may have remained higher than in PPP due to the protection effect of the microencapsulation. Flavanols including (+)‐catechin and an unidentified flavanol detected at 280 nm were quantified (20.88 ± 0.36 and 6.00 ± 0.17 mg/g dry extract, respectively) in MPP, and their content was higher than the corresponding content (8.36 ± 1.21 and 2.28 ± 0.46 mg/g dry extract) in PPP (Table 2). The flavanols found in GSE were mainly (+)‐catechin, (−)‐epicatechin, and two unknown compounds detected at 280 nm. Fruit flavanols have been demonstrated to have high efficacy in preventing lipid peroxidation in different systems and have shown positive effects against developing diseases such as atherosclerosis and coronary heart disease (Panche et al., 2016).

**TABLE 2:** Phenolic compound analysis of PPP, MPPP, and GSE^*^.

Sample	Tannins (mg/g extract)				Monomeric phenolic compounds (mg/g extract)				
	α‐Punicalagin	β‐Punicalagin	Ellagic acid	Total tannin content	Gallic acid	(+)‐catechin	(−)‐epicatechin	Unknown	Unknown
PPP	108.29 ± 34.87^b^	101.01 ± 35.68^a^	73.81 ± 28.29^a^	283.09 ± 98.81^a^	20.55 ± 2.34^b^	8.36 ± 1.21^b^	−	2.28 ± 0.46^c^	−
MPP	174.48 ± 13.01^a^	138.54 ± 9.42^a^	114.54 ± 6.40^a^	427.74 ± 28.53^a^	30.48 ± 0.20^a^	20.88 ± 0.36^a^	−	6.00 ± 0.17^a^	−
GSE	−	−	−	348.53 ± 173.09^a^	−	2.63 ± 0.21^c^	2.63 ± 0.12	3.03 ± 0.06^b^	12.73 ± 0.40

*Note*: Means with the same superscript letter in the same column are not significantly different (*p* < .05). See Table 1 for the description of PPP, MPP, and GSE.

^*^ Values are the mean ± SD of triplicate determinations.

### 3.2 Rheological properties of PPP suspensions

A non‐Newtonian, shear‐thinning (pseudoplastic) behavior (indicated by n < 1) was observed for PPP suspensions, which might be attributed to the physical disruption of chain entanglements in pectin molecules (Morales‐Contreras et al., 2018). The shear‐thinning property of PPP helped to provide a pleasant mouthfeel (Chen et al., 2020). The results also revealed that the power law model effectively described the flow behavior of PPP suspensions (*R* ^2^ = 0.996) with consistency index (*K*) value = 0.059, indicating a relatively low viscosity. Therefore, the PPP suspensions can be easily pumped and atomized into the spray dryer's chamber to produce MPP powders.

### 3.3 Emulsifying capacity and emulsion stability of ISDs

Emulsifying capacity refers to the ability of surfactants and other ingredients to facilitate the formation of food emulsions (Liang et al., 2015). ISDs homogenized at high shear rates had significantly higher (*p* < .05) emulsifying capacity (%) values than those homogenized at a lower shear rate (Table 3). At higher shear rates, the particle–particle collisions and interactions were higher, which might have resulted in smaller micelles and suspended solids. Therefore, only suspended and tiny micelles and particles remained in the salad dressings, resulting in higher emulsion capacity (Brewer et al., 2016). Interestingly, LMPP and HMPP, which contained microencapsulated PPP powders, had significantly (*p* < .05) higher EC than the other ISDs prepared with other antioxidants and homogenized at low and high shear rates, respectively (Table 3). This effect may be due to the higher viscosities and emulsification properties of the maltodextrin–pectin found in the MPP powders. Different food products show different levels of EC and ES. Although there is no defined value for EC or ES for ISDs, Fernandes and Salas Mellado (2018) reported that mayonnaises prepared with different levels of freeze‐dried chia mucilage with an EC of 63.7%. Similarly, Dabbour et al. (2018) evaluated the EC of food emulsions containing soybean oil and sunflower meal protein, and the results ranged from 49.09 to 52.45%.

**TABLE 3:** Emulsifying capacity and emulsion stability of ISDs^*^

ISD	Emulsifying capacity (%)	Emulsion stability (%)
LC	53.54 ± 0.06^d^	55.45 ± 0.26^d^
LPPP	53.96 ± 0.08^d^	55.61 ± 0.05^d^
LMPP	57.50 ± 0.13^b^	58.82 ± 0.13^c^
LGSE	53.84 ± 0.07^d^	55.68 ± 0.28^d^
HC	56.80 ± 0.14^c^	60.06 ± 0.06^b^
HPPP	57.40 ± 0.29^b^	60.22 ± 0.05^b^
HMPP	60.41 ± 0.32^a^	63.17 ± 0.05^a^
HGSE	57.44 ± 0.31^b^	60.21 ± 0.02^b^

*Note*: Means with the same superscript letter in the same column are not significantly different (*p* < .05). See Table 1 for description of LC, LPPP, LMPP, LGSE, HC, HPPP, HMPP, and HGSE.

^*^ Values are the mean ± SD of triplicate determinations.

On the other hand, emulsion stability measures the ability of food emulsions to stabilize the fine droplets during and after the emulsification process (Liang et al., 2015). The results obtained in this study showed that the emulsion stability of ISDs ranged from 55.45 to 63.17%. As in the previous case of EC, ISDs homogenized at high shear rates had significantly (*p* < .05) higher ES values than those homogenized at lower shear rates (Table 3). Moreover, LMPP had a significantly (*p* < .05) higher ES compared to LC, LPPP, and LGSE; while HMPP showed an ES (%) of 63.17 which was significantly (*p* < .05) higher than those of HC (60.06), HPPP (60.22), and HGSE (60.21) (Table 3). The higher ES values of LMPP and HMPP may be explained by the presence of maltodextrin: pectin in MPP, which may have helped create more stable suspensions with higher viscosities. Similar findings have been reported by Perrechil et al. (2010) for ES of commercial Italian salad dressings during 6 days of storage (50–65%). However, our results were lower than the ES values (81.8–88.2%) reported by Mohamad et al. (2019) who utilized cocoa butter as a stabilizer for salad dressings. According to Lozano‐Gendreau and Vélez‐Ruiz (2019), food emulsions and suspensions with high oil content (>50% w/w) may show lower values of EC and ES.

### 3.4 Lipid oxidation in ISDs under accelerated storage conditions

#### 3.4.1 Changes in pH and color

**FIGURE 2:**
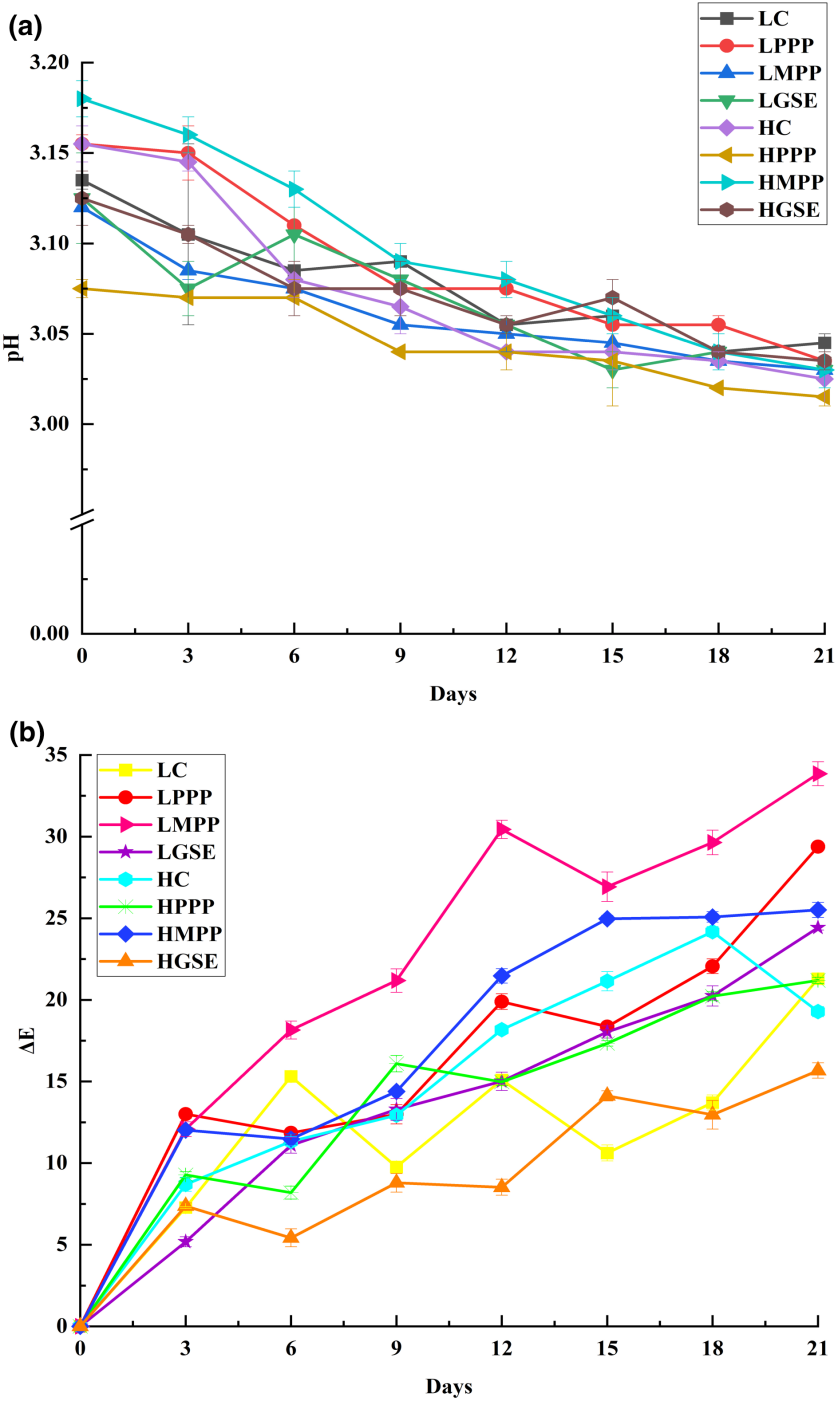
pH values (a) and color changes (b) of ISDs stored under ACSL conditions. See Table 1 for description of LC, LPPP, LMPP, LGSE, HC, HPPP, HMPP, and HGSE.

Fresh ISDs had an initial pH of ~3.08–3.16, which decreased over time and showed an average value of 3.05 at 21 days of accelerated storage (Figure 2a). It has been reported that the slight reduction in pH in salad dressing during accelerated storage may be due to increased vibrations of molecules at higher temperatures and the formation of secondary products such as acetic and propanoic acids as well as free fatty acids from lipid oxidation (Kiokias et al., 2016). According to Tseng & Zhao, 2013, the relatively stable acidic environment of salad dressings may help to stabilize polyphenols which may be able to control lipid oxidation for longer periods.

The color parameters (L*, a*, and b* values) of ISD dressings during ACSL are listed in Table 4. On day 0, all of the salad dressings had a lemon‐yellow color (hue angles between 58 and 71) with color saturation ranging from 23.29 to 32.61. An analysis of variance revealed that the antioxidants and the shear rate of homogenization had a significant effect (*p* < .05) on the color parameters of salad dressings. After 21 days of accelerated storage, the lightness (L*) and yellowness (b*) of all ISDs were significantly (*p* < .05) reduced and resulted in darker ISDs. It has been suggested that these color changes in food emulsions/suspensions with high oil concentrations may be due to flocculation which is accelerated by the lower viscosities of the continuous phase at higher storage temperatures (Lozano‐Gendreau and Vélez‐Ruiz, 2019). Interestingly, the changes in a* values (redness) of ISDs were less noticeable than the changes in L* and b* values. Furthermore, not all ISDs had a significant reduction in redness (a*) and the minor decrease in a* values could be explained by the degradation of functional ingredients due to oxidative reactions observed at high storage temperatures (Phisut et al., 2018). Moreover, the total color difference (*ΔE*) of ISDs is presented in Figure 2b. It was noted that the *ΔE* of all salad dressings was >10. LMPP had the most dramatic *ΔE* values while HGSE had the lowest value of *ΔE*. In general, salad dressings homogenized at high shear rates had lower *ΔE* than those prepared at low shear rates. The significant color differences through storage could be attributed to (a) the flocculation of the oil droplets and suspended solids (Lozano‐Gendreau and Vélez‐Ruiz, 2019); and (b) the presence of weak acids (vinegar) could lead to the extraction of more and different pigments from the ingredients at elevated temperatures which may have increased the diffusion rate and solubility of pigments in salad dressings (Mohamed et al., 2016; Oancea et al., 2012).

**TABLE 4:** Color values (L* a* b*) of Italian salad dressings (ISDs) during accelerated storage (ACSL)^a^.

Color parameter	ISD	Day 0	Day 3	Day 6	Day 9	Day 12	Day 15	Day 18	Day 21
L*	LC	21.55 ± 0.56^a^	20.67 ± 0.3^a^	17.9 ± 0.25^b^	16.58 ± 0.08^c^	14.12 ± 0.56^d^	13.07 ± 0.15^e^	11.84 ± 0.32^f^	8.21 ± 0.30^g^
	LPPP	21.59 ± 0.41^a^	19.97 ± 0.49^b^	18.46 ± 0.52^c^	18.60 ± 0.58^c^	16.94 ± 0.58^d^	13.97 ± 0.31^e^	8.76 ± 0.16^f^	5.33 ± 0.25^g^
	LMPP	26.07 ± 0.10^a^	25.86 ± 0.39^a^	22.28 ± 0.38^c^	23.66 ± 0.54^b^	17.41 ± 0.39^d^	16.05 ± 0.02^e^	13.75 ± 0.37^f^	9.66 ± 0.54^g^
	LGSE	29.94 ± 0.39	24.79 ± 0.19^b^	19.72 ± 0.44^c^	16.91 ± 0.48^d^	16.05 ± 0.33^d^	12.30 ± 0.09^e^	9.97 ± 0.39^f^	7.25 ± 0.08^g^
	HC	32.02 ± 0.14^a^	24.50 ± 0.57^b^	21.45 ± 0.32^c^	21.23 ± 0.26^c^	17.43 ± 0.08^d^	12.54 ± 0.53^f^	10.61 ± 0.24^g^	13.65 ± 0.36^e^
	HPPP	30.33 ± 0.24	23.50 ± 0.19^b^	24.49 ± 0.48^b^	15.07 ± 0.55^d^	20.00 ± 0.41^c^	13.77 ± 0.15^e^	11.70 ± 0.36^f^	11.67 ± 0.35^f^
	HMPP	35.85 ± 0.36^a^	24.14 ± 0.44^c^	26.35 ± 0.23^b^	23.90 ± 0.28^c^	16.93 ± 0.24^d^	11.47 ± 0.20^e^	11.44 ± 0.55^e^	11.07 ± 0.27^e^
	HGSE	27.54 ± 0.43^a^	26.12 ± 0.37^b^	22.61 ± 0.53^c^	21.12 ± 0.34^d^	19.07 ± 0.19^e^	14.65 ± 0.25^f^	15.04 ± 0.12^f^	11.93 ± 0.15^g^
a*	LC	15.25 ± 0.53^a^	11.06 ± 0.35^c^	9.10 ± 0.23^d^	12.2 ± 0.30^b^	9.33 ± 0.12^d^	14.75 ± 0.38^a^	15.32 ± 0.40^a^	12.51 ± 0.27^b^
	LPPP	15.26 ± 0.19^a^	13.61 ± 0.43^c^	14.76 ± 0.33^ab^	14.28 ± 0.46^b^	15.72 ± 0.15^a^	8.93 ± 0.16^f^	12.92 ± 0.42^d^	10.59 ± 0.30^e^
	LMPP	15.26 ± 0.19^a^	15.61 ± 0.22^a^	15.49 ± 0.25^a^	15.56 ± 0.28^a^	12.92 ± 0.08^b^	9.38 ± 0.28^c^	8.29 ± 0.22^d^	7.56 ± 0.57^de^
	LGSE	16.16 ± 0.37^a^	15.92 ± 0.08^a^	12.60 ± 0.15^c^	13.99 ± 0.55^b^	16.56 ± 0.05^a^	13.17 ± 0.39^b^	16.48 ± 0.42^a^	8.65 ± 0.29^d^
	HC	9.23 ± 0.16^d^	12.75 ± 0.42^a^	11.19 ± 0.14^b^	10.20 ± 0.18^c^	6.53 ± 0.19^f^	8.86 ± 0.59^e^	9.31 ± 0.43^d^	9.63 ± 0.25^d^
	HPPP	11.44 ± 0.12^b^	11.01 ± 0.57^b^	7.72 ± 0.34^e^	11.68 ± 0.12^b^	8.46 ± 0.31^d^	10.64 ± 0.32^c^	12.31 ± 0.10^a^	7.97 ± 0.17^e^
	HMPP	9.19 ± 0.19^b^	8.43 ± 0.29^bc^	8.99 ± 0.06^b^	7.54 ± 0.53^c^	5.44 ± 0.34^d^	11.94 ± 0.24^a^	7.06 ± 0.26^c^	8.88 ± 0.49^b^
	HGSE	10.38 ± 0.40^b^	7.11 ± 0.53^d^	8.62 ± 0.24^c^	11.50 ± 0.22^a^	9.82 ± 0.20^b^	7.55 ± 0.31^d^	10.63 ± 0.18^ab^	10.35 ± 0.26^b^
b*	LC	28.6 ± 0.41^a^	22.72 ± 0.49^b^	15.07 ± 0.19^f^	20.78 ± 0.39^c^	16.89 ± 0.14^e^	22.68 ± 0.59^b^	18.97 ± 0.64^d^	12.27 ± 0.54^g^
	LPPP	32.69 ± 0.46^a^	19.90 ± 0.56^c^	21.28 ± 0.44^b^	19.93 ± 0.21^c^	13.22 ± 0.26^f^	17.06 ± 0.09^d^	14.90 ± 0.25^e^	8.67 ± 0.33^g^
	LMPP	32.69 ± 0.46^a^	29.38 ± 0.45^b^	23.70 ± 0.35^c^	20.41 ± 0.15^d^	12.49 ± 0.30^h^	17.58 ± 0.51^e^	15.86 ± 0.22^f^	13.32 ± 0.47^g^
	LGSE	15.50 ± 0.53^b^	15.20 ± 0.07^b^	14.67 ± 0.32^bc^	17.02 ± 0.40^a^	9.86 ± 0.48^g^	13.30 ± 0.13^d^	12.44 ± 0.58^e^	10.53 ± 0.47^f^
	HC	27.18 ± 0.03^b^	29.74 ± 0.40^a^	23.59 ± 0.32^c^	20.07 ± 0.41^e^	16.71 ± 0.48^g^	18.77 ± 0.45^f^	15.96 ± 0.36^g^	21.36 ± 0.51^d^
	HPPP	27.10 ± 0.20^a^	20.85 ± 0.46^c^	22.75 ± 0.38^b^	22.02 ± 0.45^b^	16.69 ± 0.42^d^	22.06 ± 0.18^b^	19.31 ± 0.36^c^	17.54 ± 0.35^d^
	HMPP	23.64 ± 0.40^a^	20.87 ± 0.16^b^	17.16 ± 0.09^d^	15.79 ± 0.45^e^	13.68 ± 0.29^f^	18.82 ± 0.29^c^	23.37 ± 0.49^a^	17.48 ± 0.51^d^
	HGSE	20.05 ± 0.42^b^	13.61 ± 0.28^f^	18.79 ± 0.11^c^	25.94 ± 0.57^a^	20.46 ± 0.27^b^	15.12 ± 0.49^e^	16.15 ± 0.32^d^	18.75 ± 0.26^c^

*Note*: Means with the same letter in the same row are not significantly different (*p* < .05). See Table 1 for description of LC, LPPP, LMPP, LGSE, HC, HPPP, HMPP, and HGSE.

^a^ Values are the mean ± SD of triplicate determinations.

#### 3.4.2 Lipid oxidation

**FIGURE 3:**
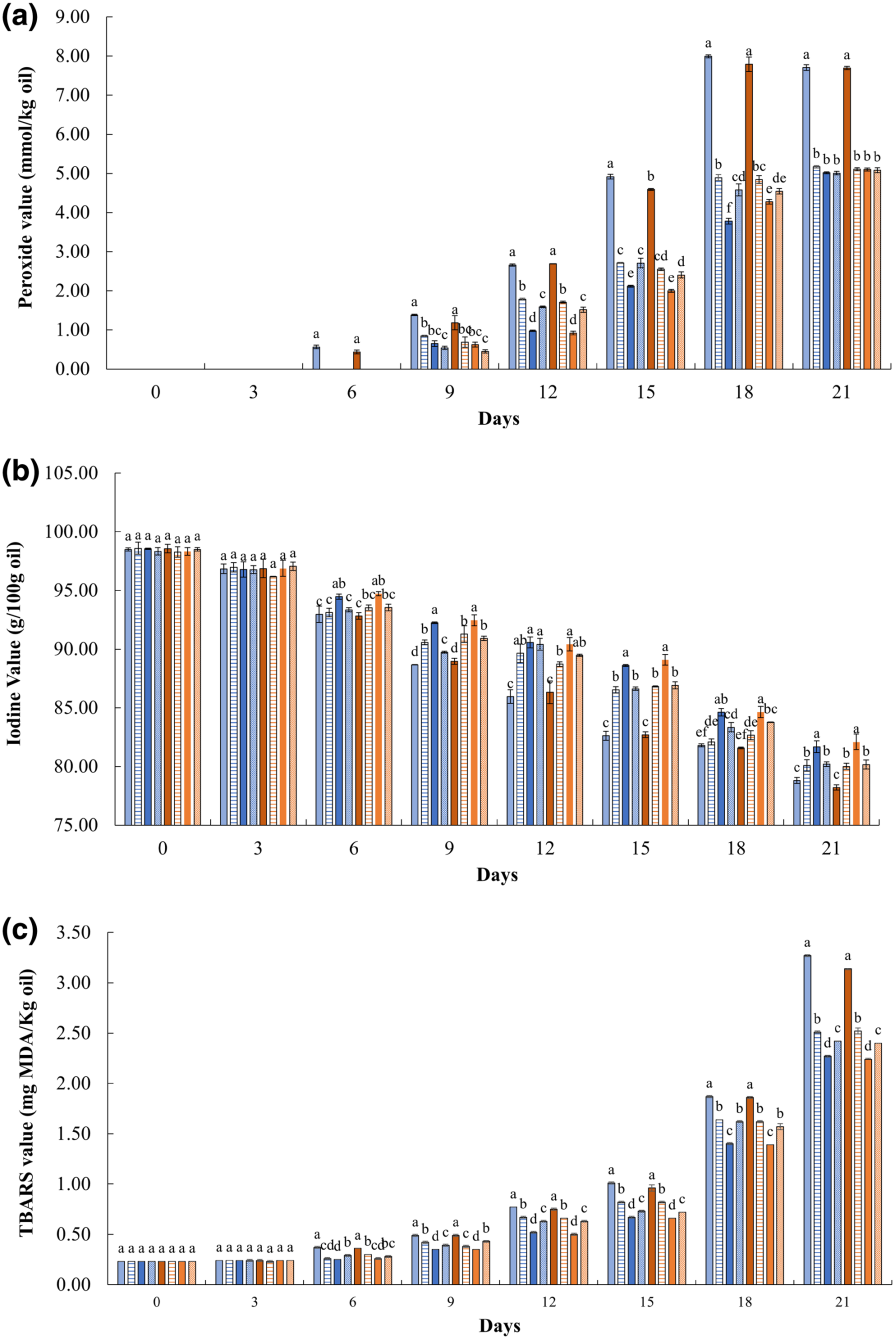
Peroxide value (a), Iodine value (b), and TBARS values (c) of Italian salad dressings (ISDs) during accelerated storage (ACSL). (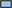 = LC; 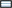 = LPPP; 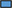 = LMPP; 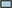 = LGSE; 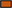 = HC; 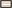 = HPPP; 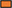 = HMPP; 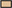 = HGSE). ^a–d^Means with the same letter in the same day are not significantly different (*p* < .05). See Table 1 for a description of LC, LPPP, LMPP, LGSE, HC, HPPP, HMPP, and HGSE.

Lipid oxidation is one of the significant concerns in food quality deterioration. The oxidative process of lipids may be catalyzed by light, heat, enzymes, metals, and microorganisms (Tseng & Zhao, 2013). PV, IV, and TBARS values are three common indicators of lipid oxidation in foods. Furthermore, PV indicates the quantity of peroxides and hydroperoxides formed in the initiation stage of lipid oxidation. As shown in Figure 3a, the PV of all ISDs significantly (*p* < .05) increased during storage, especially for those without antioxidants (LC and HC). Peroxides were detected after 6 days in LC and HC and after 9 days in antioxidant‐containing ISDs. Moreover, LC and HC had significantly (*p* < .05) higher PVs (approximately 50%) than the rest of ISDs after 21 days of storage. Curiously, the shear rates did not affect the PV of ISDs (Figure 3a). All antioxidant‐containing ISDs showed similar PVs at the end of 21 days of storage. The results may have indicated that high storage temperature could accelerate the oxidation of oils. Interestingly, antioxidants used in the study were effective at delaying lipid oxidation in ISDs to some extent. It has been reported that the PV of commercial salad dressings should be at most 10 mmol/kg oil (Lozano‐Gendreau and Vélez‐Ruiz, 2019). All ISDs were under the maximum limit for PV; however, they all showed signs of lipid oxidation, as observed in IV and TBARS. Vegetable oils with a high content of polyunsaturated fatty acid (PUFA) are more vulnerable to lipid oxidation, while the presence of saturated fatty acid (SFA) and monounsaturated fatty acid (MUFA) could improve their oxidative stability (Cao et al., 2015). The main fatty acids in peanut oil are oleic acid (45–53%, MUFA), linoleic acid (27–32%, PUFA), and palmitic acid (11–14%, SFA) (Ghazani & Marangoni, 2016). PV can only measure initial lipid oxidation products; meanwhile, hydroperoxides are unstable molecules that decompose quickly into secondary oxidation products such as aldehydes during storage at elevated temperatures (Eidhin & O'Beirne, 2010). This may have occurred in LH and HC after day 18 (the PVs of LC and HC at day 18 were higher than those of corresponding samples on day 21). Given the possibility of decomposition of hydroperoxides, PV only was not enough to assess the quality of edible oils.

The iodine value measures the degree of unsaturation of fatty acids. A decrease in IV indicates an increase in the degree of saturation of fatty acids (Ayodeji & Ganiyu, 2015). Changes in IV of ISDs are presented in Figure 3b. During the first 12 days of storage, all ISDs were within the standard range (84–107) of fresh peanut oil (Karl, 2017). Not surprisingly, the IV of LC and HC reduced at higher rates than those of antioxidant‐containing ISDs. After 21 days of storage, the IV of LC and HC was significantly (*p* < .05) lower than the rest of the ISDs. Interestingly, LMPP and HMPP had significantly (*p* < .05) higher IV (+5%) than those of LC and HC after 21 days of storage. Even more, shear rates did not have any effect on the IV of the ISDs during ACSL. These results suggested that ISDs experienced a reduction in the unsaturation of their fatty acids due to the breakdown of carbon chain bindings, thus forming saturated carbon chains (Mohamad et al., 2019). Antioxidants containing ISDs, especially those containing MPPP powders, have shown lower lipid oxidation than ISDs without antioxidants (especially in the first 15 days of storage). Similar trends for IV have been reported by Guo et al. (2016) in palm oil with rosemary ethanol extract during frying and accelerated storage and Jahurul et al. (2017) in mango seed fat and palm oil mid‐fraction blends as cocoa butter replacers under accelerated storage conditions.

The TBARS is a parameter used to monitor the production of secondary lipid oxidation products, mainly malondialdehyde (MDA). It was noted that the TBARS of all ISDs significantly (*p* < .05) increased after 21 days of storage (Figure 3c). Surprisingly, LMPP and HMPP showed significantly (*p* < .05) lower TBARS than the rest of the treatments after 21 days of storage. Furthermore, there was no apparent effect of the shear rates on the TBARS values of ISDs. Food products with TBARS values lower than 0.576 mg MDA/kg dry weight (DW) of the sample are considered fresh, those with TBARS values between 0.65 and 1.44 mg MDA/kg DW are considered rancid but still acceptable, and those with TBARS values higher than 1.5 mg MDA/kg DW are considered unacceptable for consumption (Cong et al., 2020; Fuchs et al., 2015; Singh et al., 2020). Using those criteria, all treatments were considered fresh after 9 days of storage. After 18 days of accelerated storage, all ISDs, but LMPP and HMPP, could have been considered unacceptable for consumption. In addition, the results confirmed that antioxidants effectively delayed the formation of MDA in salad dressings (Phisut et al., 2018). Also, these findings suggest that MPP was an effective antioxidant for delaying lipid oxidation in a salad dressing system after 21 days of accelerated storage and that the breakdown of peroxides to carbonyl and aldehyde compounds such as MDA was accelerated by high storage temperatures (Ayodeji & Ganiyu, 2015).

Accelerated storage is always a cost‐effective approach when predicting the shelf life of foods (Feng, 2011). The estimated shelf life can be calculated based on Equation (6) (Joseph, 2016):

(6) $$\text{Estimated shelf life}={Q_{10}}^{\left(T1-T2/10\right)}\times \text{days of ACSL}$$

where *Q*_10_ is a typical value (2.0) to estimate reaction rates in food, *T*_1_ is the temperature (55°C) of accelerated conditions, and *T*_2_ is the room temperature (25°C).

In this study, 21 days of ACSL was equivalent to 168 days (5.6 months) of ambient storage. Using PV as an indicator for the determination of the shelf life of ISDs, all treatments were within the normal range after 21 days of ACSL. Furthermore, if the results for IV were to be used to calculate shelf life, the IVs of LC and HC were below the normal range after 15 days of accelerated storage (equivalent to 120 days of ambient storage), while MPP‐containing salad dressings were still acceptable after 18 days of ACSL. Using TBARS as the leading indicator, the LC, LPPP, LGSE, HC, HPPP, and HGSE treatments were considered rancid and unacceptable after 15 days of accelerated storage (equivalent to 120 days of ambient storage), while LMPP and HMPP were still considered acceptable after 18 days (equivalent to 144 days of ambient storage). These results suggest that MPP may extend the shelf life of ISDs by 24 days by delaying lipid oxidation. However, the results obtained under accelerated shelf life studies must be interpreted with care when predicting the shelf life of foods. Because the oxidation mechanisms could change with temperature, samples could exhibit excessive rancidity, which is not associated with typical storage conditions. Depending on the type of oil, these predictions may lead to an overestimation or underestimation of the actual shelf life (Farhoosh, 2007). Therefore, it is recommended to confirm accelerated storage with ambient storage conditions. Nevertheless, when time is constrained, accelerated storage studies provide an exciting approach to evaluating the preliminary effectiveness of natural antioxidants. A similar study by Aksoy et al. (2022) reported the use of OXITEST to evaluate the oxidative stability of salad dressings enriched with microencapsulated phenolic extracts from cold‐pressed grape and pomegranate seed oil by‐products. The study demonstrated that incorporating those natural antioxidants significantly increased the oxidation stability of the salad dressings without lowering their physical stability.

### 3.5 Lipid oxidation in ISDs during ambient storage conditions

#### 3.5.1 Changes in pH and color

**FIGURE 4:**
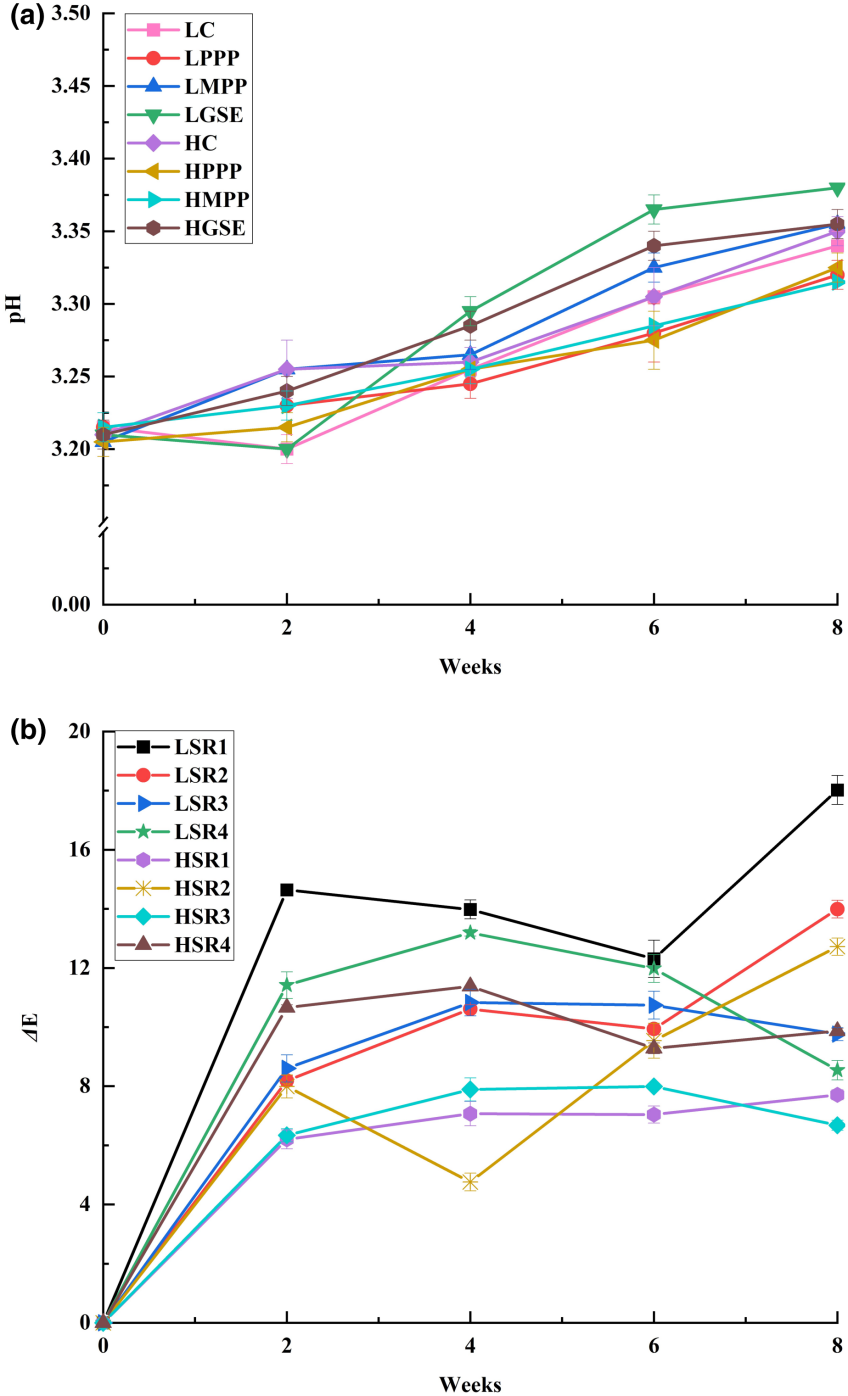
pH values (a) and color changes (b) of ISDs during ambient storage conditions. See Table 1 for description of LC, LPPP, LMPP, LGSE, HC, HPPP, HMPP, and HGSE.

The pH of the aqueous phase has been reported as a critical factor in controlling the microstructural stability of food emulsions and suspensions (Seo et al., 2013). The pH changes of salad dressings during ambient storage conditions are presented in Figure 4a. The initial pH values of ISDs were ~3.2 and significantly (*p* < .05) increased to 3.32–3.38 after storage (25°C, relative humidity of 40–60% in the dark) for 8 weeks. The increased pH might be explained by the slight decomposition of acetic acid and other ingredients in the salad dressings during storage in a warm and humid environment (Ahmad, 2020). According to Park and Lee (2009), acetic acid can be decomposed in oxidation processes in the presence of ultraviolet radiation. During storage, the oxygen from the air can slowly diffuse into the salad dressings, leading to oxidative reactions. The oxidative degradation of acetic acid may have caused the increase in pH. Additionally, microorganisms present in the salad dressing can potentially metabolize acetic acid as a carbon source, leading to its decomposition.

Generally, the pH of salad dressings is <4.6 (pH of acidified foods) which limits microbial growth during storage at ambient temperature (Breidt et al., 2014).

The color parameters (L*, a*, and b* values) of salad dressings during ambient storage are presented in Table 5. Fresh ISDs had different lightness, redness, and yellowness because of different homogenization conditions and different types of ingredients used. After 8 weeks of storage under AMSL conditions, the degree of lightness of all salad dressings was significantly (*p* < .05) lower than that of the corresponding fresh samples while there were no obvious patterns of change in the values of a* and b* during storage. These results confirmed the results observed in ACSL, where darker ISDs were observed after 21 days of storage.

**TABLE 5:** Color values (L* a* b*) of Italian salad dressings (ISDs) stored under ambient storage (AMSL) conditions^a^.

Color value	ISD	Week 0	Week 2	Week 4	Week 6	Week 8
L*	LC	18.65 ± 0.13^a^	10.72 ± 0.24^d^	11.43 ± 0.20^b^	14.38 ± 0.31^b^	9.50 ± 0.07^e^
	LPPP	18.95 ± 0.08^a^	13.56 ± 0.23^e^	14.82 ± 0.37^d^	16.05 ± 0.11^c^	17.50 ± 0.13^b^
	LMPP	25.03 ± 0.12^a^	17.58 ± 0.14^b^	14.75 ± 0.22^e^	19.65 ± 0.15^b^	15.84 ± 0.31^d^
	LGSE	20.95 ± 0.43^a^	17.00 ± 0.14^b^	15.10 ± 0.23^c^	17.01 ± 0.06^b^	16.62 ± 0.30^b^
	HC	18.95 ± 0.13^a^	14.60 ± 0.36^c^	17.53 ± 0.16^b^	17.32 ± 0.19^b^	15.01 ± 0.13^c^
	HPPP	19.83 ± 0.10^a^	17.49 ± 0.18^c^	16.82 ± 0.33^d^	18.55 ± 0.03^b^	18.32 ± 0.09^b^
	HMPP	23.23 ± 0.11^a^	19.46 ± 0.36^b^	16.82 ± 0.42^d^	19.51 ± 0.19^b^	18.58 ± 0.22^c^
	HGSE	25.91 ± 0.15^a^	15.93 ± 0.03^c^	14.62 ± 0.04^d^	17.34 ± 0.06^b^	16.19 ± 0.07^c^
a*	LC	12.63 ± 0.34^c^	15.93 ± 0.21^a^	14.25 ± 0.20^b^	9.42 ± 0.22^d^	14.37 ± 0.36^b^
	LPPP	12.07 ± 0.26^a^	12.69 ± 0.23^a^	9.58 ± 0.28^b^	9.28 ± 0.11^b^	9.40 ± 0.17^b^
	LMPP	9.06 ± 0.29^b^	9.24 ± 0.31^b^	8.16 ± 0.13^c^	6.61 ± 0.24^d^	9.97 ± 0.31^a^
	LGSE	13.77 ± 0.27^a^	9.51 ± 0.27^c^	9.33 ± 0.10^c^	10.94 ± 0.30^b^	13.22 ± 0.51^a^
	HC	9.40 ± 0.14^ab^	8.89 ± 0.57^b^	7.62 ± 0.04^c^	7.08 ± 0.12^c^	9.66 ± 0.10^a^
	HPPP	9.88 ± 0.11^a^	8.30 ± 0.01^b^	9.43 ± 0.32^a^	7.94 ± 0.16^b^	8.05 ± 0.10^b^
	HMPP	12.39 ± 0.12^a^	11.03 ± 0.14^b^	11.53 ± 0.28^b^	10.00 ± 0.37^c^	12.51 ± 0.21^a^
	HGSE	12.64 ± 0.21^b^	13.94 ± 0.25^a^	12.11 ± 0.27^b^	12.86 ± 0.24^b^	13.99 ± 0.11^a^
b*	LC	30.07 ± 0.71^a^	18.23 ± 0.40^b^	18.21 ± 0.19^b^	18.98 ± 0.17^b^	14.64 ± 0.33^c^
	LPPP	27.95 ± 0.19^a^	21.83 ± 0.33^b^	18.51 ± 0.20^c^	18.86 ± 0.24^c^	14.29 ± 0.53^d^
	LMPP	22.02 ± 0.92^a^	18.10 ± 0.64^b^	16.27 ± 0.17^c^	13.07 ± 0.45^d^	18.91 ± 0.49^b^
	LGSE	25.37 ± 0.60^a^	15.55 ± 0.52^d^	14.43 ± 0.25^e^	19.40 ± 0.58^b^	18.04 ± 0.35^c^
	HC	26.44 ± 0.28^a^	22.07 ± 0.10^b^	19.75 ± 0.18^d^	20.01 ± 0.16^c^	19.82 ± 0.53^cd^
	HPPP	28.14 ± 0.82^a^	20.66 ± 0.49^c^	24.49 ± 0.76^b^	18.89 ± 0.21^d^	15.65 ± 0.64^e^
	HMPP	27.94 ± 0.48^a^	22.79 ± 0.40^bc^	23.44 ± 0.44^b^	21.29 ± 0.38^c^	23.15 ± 0.59^b^
	HGSE	20.41 ± 0.47^c^	23.71 ± 0.26^a^	20.43 ± 0.47^c^	23.95 ± 0.28^a^	21.44 ± 0.46^b^

*Note*: Means with the same letter in the same row are not significantly different (*p* < .05). See Table 1 for description of LC, LPPP, LMPP, LGSE, HC, HPPP, HMPP, and HGSE.

^a^ Values are the mean ± SD of triplicate determinations.

The total color difference (*ΔE*) of ISDs during AMSL is shown in Figure 4b. In general, ISDs prepared at high shear rates showed lower changes in color compared to the ISDs prepared at low shear rates. Furthermore, compared with *ΔE* of salad dressing during accelerated storage, the *ΔE* of all treatments was smaller under ambient storage. Moreover, the *ΔE* of HC and HMPP were relatively low and stable (6–8) during the 8‐week storage period, which indicated that their color did not change as much as in the case of the other ISDs. It has been hypothesized that the different ΔE values could be explained by the extrinsic color changes of ingredients as well as the homogenization conditions such as shear rates and homogenization time (Eissa et al., 2016). Overall, these color parameters should be taken into account when formulating various salad dressings because consumers have a preconceived prospect of the appearance of the different products (Chung et al., 2017).

#### 3.5.2 Lipid oxidation

**FIGURE 5:**
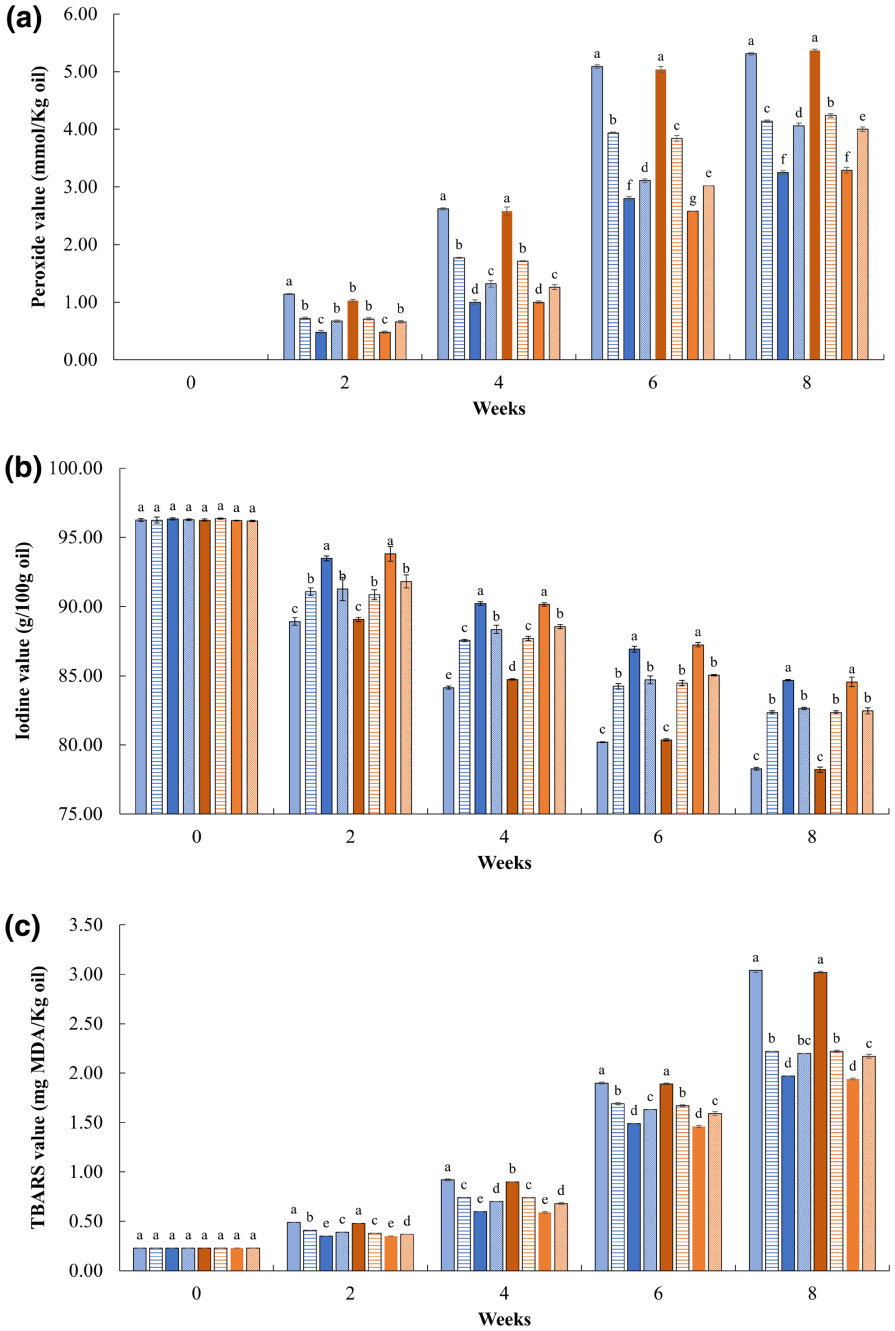
Peroxide value (a), iodine value (b), and TBARS values (c) of Italian salad dressings (ISDs) during ambient storage (AMSL). (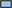 = LC; 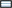 = LPPP; 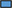 = LMPP; 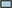 = LGSE; 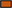 = HC; 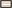 = HPPP; 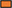 = HMPP; 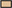 = HGSE). ^abcd^Means with the same letter in the same day are not significantly different (*p* < .05). See Table 1 for description of LC, LPPP, LMPP, LGSE, HC, HPPP, HMPP, and HGSE.

As previously stated, the auto‐oxidation of oil is a major problem in salad dressings, and the primary products from lipid oxidation can be measured as PV (Kishk & Elsheshetawy, 2013). The PV of ISDs under AMSL is shown in Figure 5a. It was observed that all ISDs started to show signs of oxidation within the first 2 weeks of storage. Also, all samples showed PV lower than 5.5 mmol/kg oil after 8 weeks of storage. Interestingly, LMPP and HMPP had significantly (*p* < .05) lower PVs than the rest of the ISDs at the end of the storage time. The results also revealed that ISDs prepared with MPP had the lowest PV followed by those prepared with GSE and PPP, respectively. It appears that shear rates did not affect the PV of ISDs under AMSL. Interesting findings were made after comparing PV in ACSL (Figure 3a) with PV in AMSL (Figure 5a). PVs obtained in ACSL (3 weeks) were higher than those obtained under AMSL (8 weeks). This finding was consistent with the previous findings that PVs under ambient storage were lower than those obtained under ACSL (Branco et al., 2011). Similar results have been reported by Mohammadi et al. (2016), who demonstrated that microencapsulation of phenolic compounds in double emulsion systems can increase antioxidant capacity due to a controlled release effect. An antioxidant's relative effectiveness depended on the lipid substrate, physical state (emulsion), oxidation time, and temperature (Lee et al., 2014).

The IVs of all salad dressings during ambient storage are presented in Figure 5b. Initially, all ISDs had IVs higher than 96 g iodine/100 g oil. Then, the IV of all ISDs was significantly (*p* < .05) reduced during ambient storage. At the end of the 8 weeks, LMPP and HMPP showed significantly (*p* < .05) higher IVs compared to the rest of the treatments. Moreover, the IV of LMPP and HMPP was ~8.1% higher than those of LC and HC. Meanwhile, LC and HC showed the lowest IV, which may have indicated the highest decrease in unsaturation (presumably due to oxidation). As in the case of PV, there was no apparent effect of shear rates on the IV of ISDs. The results suggested that antioxidants could help to inhibit/delay the destruction of fatty acid double bonds, thus delaying lipid oxidation. In addition, the controlled release of MPP helped sustain their antioxidant activities longer than unencapsulated/free antioxidants.

The initial TBARS value of all ISDs was 0.23 mg MDA/kg oil. At the end of 8 weeks, LC and HC showed a significantly (*p* < .05) higher TBARS (~3.02–3.04 mg MDA/kg oil) than the rest of ISDs. Moreover, LMPP and HMPP showed significantly (*p* < .05) lower TBARS than the rest of the treatments (Figure 5c). As in the previous cases of PV and IV, there was not an apparent effect of shear rates on TBARS values of ISDs. As we mentioned previously, LC, LPPP, LGSE, HC, HPPP, and HGSE may have been classified as rancid but still acceptable; while LMPP and HMPP could have been classified as fresh (TBARS < 0.6 mg MDA/Kg oil) after 4 weeks of storage. After 6 weeks of storage, all ISDs, but LMPP and HMPP, could have been classified as unacceptable for consumption. It has been reported that MDA is one of the many reactive electrophile species that cause oxidative stress in cells and the formation of advanced glycation end‐products, which are associated with several degenerative diseases such as cancer, diabetes mellitus, and kidney dysfunction (Oboh et al., 2014).

Normally, polyphenols can act as chain‐breaking antioxidants, hydroperoxide destroyers, and metal chelators (Chong et al., 2015). The phenolic hydroxyl groups could donate hydrogen atoms to scavenge free radicals such as hydroxyl, peroxyl, superoxide, and nitric oxide which were produced from the mixtures of secondary oxidation products and transition metals in the aqueous phase of salad dressings, resulting in retardation of the initiation or propagation stage of lipid oxidation. Therefore, these antioxidants can interfere with further lipid oxidation in salad dressings.

## 4 CONCLUSION

The study demonstrated the effectiveness of incorporating microencapsulated polyphenols from pomegranate peels (MPP) in the Italian salad dressings system to control lipid oxidation during accelerated and ambient storage conditions. All fresh salad dressings had a lemon‐yellow color, and those prepared at high shear rates had significantly higher emulsion stability than those prepared at low shear rates. Mixing conditions did not affect the lipid oxidation and quality deterioration in the salad dressings during storage. Results from the accelerated storage suggested that incorporating MPP could have extended the shelf life of salad dressings by 20% (24 days) compared to using an unencapsulated polyphenols extract. Moreover, MPP‐containing salad dressings stored at accelerated and ambient conditions showed less indication of lipid oxidation than those prepared with unencapsulated polyphenol extract. Microencapsulation provides an exciting potential to improve the stability of natural antioxidants when they are added to high lipid content and acidified foods to control lipid oxidation.

## AUTHOR CONTRIBUTIONS

**Boran Yang:** Conceptualization (equal); investigation (lead); methodology (lead); software (equal); writing – original draft (lead). **Jinru Chen:** Funding acquisition (lead); resources (equal); writing – review and editing (equal). **Kevin Mis Solval:** Data curation (equal); funding acquisition (equal); investigation (supporting); methodology (supporting); project administration (lead); resources (equal); supervision (lead).

## FUNDING INFORMATION

This research was funded by the USDA/AMS (Agricultural Marketing Service) Specialty Crop Block Program grant (SCBGP) administered by the Georgia Department of Agriculture Award no. RSRC0001136001.

## CONFLICT OF INTEREST STATEMENT

There are none to declare.

## Data Availability

Limited data will be made available upon request.
